# CYPOR Variability as a Biomarker of Environmental Conditions in Bream (*Abramis brama*), Roach (*Rutilus rutilus*), Perch (*Perca flavescens*), and Pike-Perch (*Sander lucioperca*) from Lake Ladoga

**DOI:** 10.3390/vetsci13010094

**Published:** 2026-01-18

**Authors:** Vladimir Ponamarev, Olga Popova, Elena Semenova, Evgeny Mikhailov, Alexey Romanov

**Affiliations:** 1 Department of the Pharmacology and Toxicology, Saint-Petersburg State University of Veterinary Medicine, Chernigovskaya St., 5, St. Petersburg 196084, Russia; 2 Laboratory of Innovative Recombinant Proteomics Medicines, All-Russian Veterinary Research Institute of Pathology, Pharmacology and Therapy, Lomonosov St., 114b, Voronezh 394087, Russia; 3 Department of the Experimental Pharmacology and Modeling of Living Systems, All-Russian Veterinary Research Institute of Pathology, Pharmacology and Therapy, Lomonosov St., 114b, Voronezh 394087, Russia; 4 Laboratory of Fisheries Ecology, All-Russian Research Institute of Fisheries and Oceanography Named After L.S. Berg, Makarova Embankment, 26, St. Petersburg 199053, Russia

**Keywords:** detoxification, xenobiotic, cytochrome P450, hepatopathies

## Abstract

The fish liver, as the main detoxification organ, is highly susceptible to xenobiotic exposure, often resulting in various hepatopathies. The cytochrome P450 system plays a central role in xenobiotic metabolism, with cytochrome P450 reductase (CYPOR) supplying the electrons required for CYP enzyme activity. This study aimed to evaluate the relationship between the ecological state of a reservoir and fish health, including CYPOR levels, through hematological, bacteriological, and histological analyses. Samples of water and fish were collected from 12 littoral sites of Lake Ladoga. A total of 1360 specimens of fish from carp (*Cyprinidae*) and perch (*Percidae*) families were examined. For histological examination and CYPOR level determination, we selected 40 specimens using a blind randomization method. This sample size was sufficient for statistical analyses. Hematological smears were stained with azure eosin; bacteriological cultures were grown on multiple media; liver samples were stained with hematoxylin and eosin and Sudan III. CYPOR levels in liver homogenates were measured by ELISA-test. Physical and hydrochemical analyses indicated a high pollution level in the littoral zones. Isolated bacterial species were non-pathogenic but exhibited broad antibiotic resistance. Hematological evaluation revealed erythrocyte vacuolization and anisocytosis. Histological analysis showed marked fatty degeneration in hepatocytes, indicating toxic damage. CYPOR concentrations ranged from 0.3–0.4 ng/mL in healthy fish to 5–6 ng/mL in exposed specimens, showing strong correlation between environmental influence and enzyme activity. These findings demonstrate the potential of CYPOR as a sensitive biomarker for biomonitoring programs. The integrated methodological approach provides a model for assessing aquatic ecosystem health and identifying zones requiring priority remediation.

## Simple Summary

Fish are valuable indicators of aquatic ecosystem health because their physiological condition reflects the quality of their habitat. The liver is particularly important, as it is responsible for detoxifying harmful substances and therefore responds sensitively to pollution. This study investigated how the ecological condition of the littoral zones of Lake Ladoga influences fish health. Forty fish belonging to the carp and perch families were collected from twelve coastal sites. Water quality was assessed, and hematological, bacteriological, and histological examinations of the fish were conducted to evaluate their health status. The results revealed that the coastal areas of Lake Ladoga are exposed to significant pollution. Although the bacterial species isolated from the fish were typical for healthy individuals, many showed resistance to several antibiotics, which is a matter of concern. The blood tests indicated structural abnormalities in red blood cells, and liver tissue showed changes consistent with toxic damage. In fish from polluted areas, the level of a key liver enzyme (CYPOR) involved in detoxification was markedly higher than in healthy fish. These findings suggest that CYPOR can serve as a sensitive biomarker for environmental stress, supporting more effective monitoring of aquatic ecosystems.

## 1. Introduction

The fish liver, as the primary detoxification organ, is the first to be adversely affected by xenobiotics, leading to the development of various forms of hepatopathies [1,2,3,4]. The lipophilic properties of many antibiotics allow them to enter the bodies of aquatic organisms through the gills and digestive tract, facilitating rapid transmembrane transport and accumulation in tissues, particularly in the liver. Agbabiaka et al. [5] reported that the concentration of antibiotics in hepatocytes may exceed their levels in the surrounding aquatic environment by 100–1000 times due to bioaccumulation. Consequently, the liver is the organ most susceptible to damage, which manifests as hepatopathies.

Hepatopathies in fish can be caused by various factors, including toxic substances, infections, and genetic predisposition [6,7]. The cytochrome P450 system plays a key role in the metabolism of xenobiotics and endogenous compounds, while cytochrome P450 reductase (CYPOR) provides the electrons required for the catalytic activity of CYP enzymes [8,9]. Disruptions in the functioning of these systems may lead to the development of hepatopathies. At present, quantitative assessment of hepatic CYPOR as a biomarker in aquatic ecotoxicology remains limited, highlighting the novelty of this study.

At the molecular level, the primary detoxification of antimicrobial pharmaceuticals in the fish liver is carried out by the cytochrome P450 (CYP) system. The predominant isoforms in fish include CYP1A, CYP2B, and CYP3A [10]. The cytochrome P450 system comprises numerous isoenzymes involved in the biotransformation of various substances. CYPOR is a flavoprotein that transfers electrons from NADPH to CYP, a process essential for CYP catalytic activity. Disruptions in CYP and CYPOR function can lead to several consequences:

1. Toxin accumulation: Insufficient CYP activity can slow the metabolism of toxic substances, promoting their accumulation in the liver and contributing to the development of hepatopathies.
2. Oxidative stress: CYPOR and CYP participate in the generation of reactive oxygen species (ROS). Impaired function of these systems can increase ROS production, leading to oxidative stress and hepatocellular damage.
3. Inflammatory processes: Metabolic products formed through CYP-mediated reactions may induce inflammatory responses that contribute to hepatopathy development.

Furthermore, the activity of cytochrome isoforms increases significantly following antibiotic exposure. The metabolism of most antimicrobial agents is accompanied by the formation of reactive intermediates capable of inducing oxidative stress [10]. Studies have shown that exposure to toxicants such as polycyclic aromatic hydrocarbons (PAHs), polychlorinated biphenyls (PCBs), and pesticides can alter the expression and activity of CYP and CYPOR in fish. Liu et al. [11] demonstrated in experiments on common carp (*Cyprinus carpio*) that exposure to subtherapeutic doses of oxytetracycline resulted in a significant increase in malondialdehyde levels and a decrease in the activity of superoxide dismutase, catalase, and glutathione peroxidase in the liver, indicating disruption of the pro- and antioxidant balance. Reactive oxygen species generated during antibiotic metabolism cause lipid peroxidation of hepatocyte membranes, leading to impaired selective permeability, damage to membrane proteins, and the release of intracellular enzymes such as ALT and AST into the bloodstream. These findings are supported by the results reported by Chen et al. and Allameh et al. [12,13].

Based on the study by Larina et al. [14] using hepatocyte cell lines from silver crucian carp (*Carassius auratus*), it can be assumed that antimicrobial agents are capable of modulating gene expression in fish hepatocytes through interactions with nuclear receptors, including the pregnane X receptor (PXR), constitutive androstane receptor (CAR), and aryl hydrocarbon receptor (AhR). Rodríguez-Mozaz et al. [15] reported that sulfonamides induce CYP1A gene expression via AhR activation, resulting in increased formation of toxic metabolites and enhanced oxidative stress. Ambili et al. [16] noted that macrolide antibiotics stimulate the production of proinflammatory cytokines (IL-1β, TNF-α) by Kupffer cells, increasing the infiltration of neutrophils and lymphocytes into the fish liver. These antibiotics also activate the NF-κB and AP-1 signaling pathways in hepatocytes, enhancing the expression of inflammatory response genes [17].

Long-term exposure to antimicrobials activates programmed cell death pathways in the fish liver. Paramonov et al. [18] reported that chronic exposure to tetracyclines in carp activates both extrinsic (via Fas receptors) and intrinsic (mitochondrial) apoptotic pathways. This exposure induces hypermethylation of promoter regions of antioxidant defense genes, resulting in reduced gene expression and increased hepatocyte sensitivity to oxidative stress. Ding et al. [19] demonstrated that antimicrobial agents can modify histone acetylation patterns in fish hepatocyte nuclei, thereby affecting the transcriptional activity of genes involved in xenobiotic metabolism. Prolonged antimicrobial exposure also contributes to liver fibrosis in fish. Studies by Au-Yeung et al. and Andreeva et al. [20,21] showed that long-term exposure of carp to sublethal concentrations of chloramphenicol activates Ito cells, which differentiate into myofibroblasts and synthesize extracellular matrix components, primarily collagen types I and III.

The aim of this study was to assess the relationship between the ecological condition of Lake Ladoga and its coastal zone, fish health status, and CYPOR levels using hematological, bacteriological, and histological analyses.

The main objectives of the study were:

1. To evaluate the ecological state of the reservoir and its coastal zone as fish habitats.
2. To conduct hematological analyses to assess the clinical status of fish.
3. To perform bacteriological and histological examinations to further evaluate fish health.
4. To determine CYPOR levels and assess the impact of habitat conditions on these parameters.

## 2. Materials and Methods

The studies were conducted at the Department of Pharmacology and Toxicology of the Federal State Budgetary Educational Institution of Higher Education St. Petersburg State University of Veterinary Medicine (St. Petersburg, Russia) and at the Laboratory of Innovative Recombinant Proteomic Preparations of the Department of Experimental Pharmacology and Modeling of Living Systems, Federal State Budgetary Scientific Institution All-Russian Veterinary Research Institute of Pathology, Pharmacology and Therapy (Voronezh, Russia).

**Figure 1:**
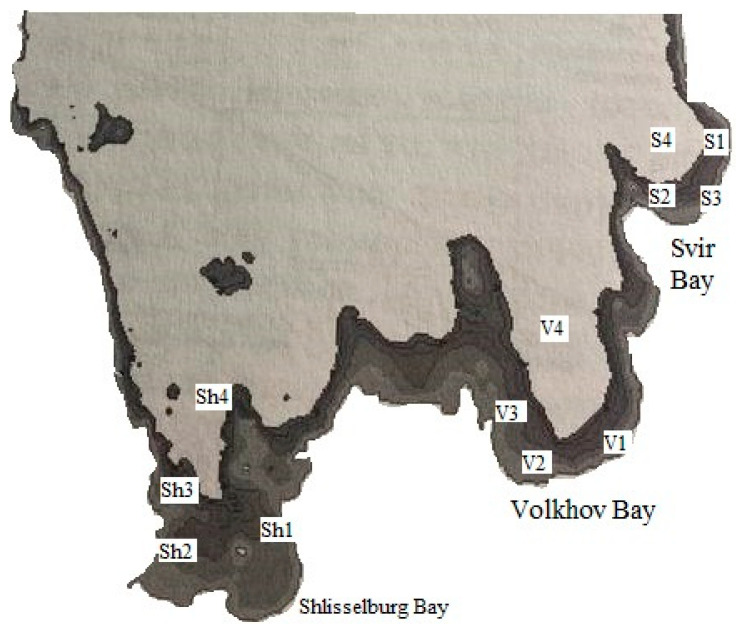
Geography of the research stations. S1, S2, S3, S4—stations near Svir bay; V1, V2, V3, V4—stations near Volkhov bay; Sh1, Sh2, Sh3, Sh4—stations near Shlisselburg bay.

For the main study, aquatic organisms were collected from Lake Ladoga, specifically from its shallow southern littoral zone, which is considered the most vulnerable to toxic impacts (Figure 1, Table 1). The water of Lake Ladoga is characterized by low mineralization, a hydrocarbonate–calcium composition, and a slight predominance of sulfate ions over chloride ions. Physical and hydrochemical parameters of water in three areas of Lake Ladoga were assessed using an Aqua TROLL 500 multiparameter probe (In-Situ, Fort Collins, CO, USA). The following parameters were measured: temperature (T, °C), mineralization (Mn), dissolved oxygen concentration (O_2_), electrical conductivity (EC), oxidation–reduction potential (ORP), and hydrogen ion concentration (pH).

Fish were sampled from the littoral zones and adjacent waters of three bays: Shlisselburg, Volkhov, and Svirskaya. A total of 1360 fish specimens belonging to the families Cyprinidae (bream (*Abramis brama*) and roach (*Rutilus rutilus*)) and Percidae (perch (*Perca flavescens*) and pike-perch (*Sander lucioperca*)) caught in the Shlisselburg, Volkhov, and Svir Bays were examined. For histological and bacteriological analyses and determination of CYPOR levels, 40 specimens (10 individuals of each species) were selected from waters near the research stations using a blind randomization method. The fish were chosen by random number generation from the total catch, with stratification by sampling site and species to ensure representativeness. These specimens constituted the experimental group. This sample size was selected for bioethical reasons, to maintain statistical significance, and to accommodate the technical capabilities of the laboratory.

For the control group, 40 clinically healthy, farm-raised fish from the *Cyprinidae* and *Percidae* families maintained under standardized and stable conditions were used. Fish were housed in 150–200 L glass aquaria at a stocking density of 1.0–1.5 kg/m^3^, with continuous aeration and mechanical–biological filtration. A natural photoperiod was maintained (10 h of fluorescent light and 14 h of darkness). Water temperature was maintained at 20–24 °C. Fish were fed a balanced diet at a daily ration of 3% of their biomass, divided into three feedings (07:00, 12:00, and 17:00), to minimize additional stress. To maintain water quality, 30% of the aquarium water volume was replaced daily, and uneaten feed and feces were removed. In the event of fish mortality, microbiological and molecular genetic analyses of pathological material were performed.

All fish manipulations were conducted in accordance with the bioethical guidelines described in the Guide for the Care and Use of Laboratory Animals [22].

### 2.1. Hematological Examination

Peripheral blood samples from common carp were collected from the caudal vein using the method described by Petrova [23]. Blood was obtained at the point of intersection of a line drawn from the posterior edge of the anal fin. Prior to sampling, scales were removed and the puncture site was disinfected with a 70% ethanol solution. The needle was inserted at a 45° angle toward the head, advanced until contact with the spinal column, and slowly withdrawn until blood appeared in the syringe cannula. After collection, the needle was removed and the puncture site was treated with an antiseptic.

Blood smears were prepared and stained using standard methods [24,25]. After air drying for 4 min, smears were fixed in Nikiforov’s solution (1:1 methanol–diethyl ether) for 2 min, stained with May–Grünwald methylene blue–eosin for 4 min, rinsed, and subsequently stained with Romanovsky–Giemsa azure–eosin for 10 min. The preparations were then washed with distilled water and air-dried for 30 min.

### 2.2. Bacteriological and Histological Studies

To determine the qualitative and quantitative composition of the microbiota, samples were cultured on standard selective and enrichment media, including meat-peptone agar, Endo agar, mannitol salt agar, blood agar (meat-peptone agar supplemented with 5% sheep erythrocytes), Kitt–Tarozzi medium, Wilson–Blair medium, Blaurock medium, and MRS agar for lactobacilli (all media: Research Center for Pharmacotherapy, Saint Petersburg, Russia). Facultative anaerobic bacteria were incubated at 37 °C under aerobic conditions, while obligate anaerobes were cultured using an anaerobic jar. For the detection of microscopic fungi, samples were incubated at 28 °C. After 18–72 h of incubation, colonies were counted, and the cultural, morphological, and biochemical properties of isolates were assessed using standard methods. Bacterial identification was performed according to Skorodumov and Subbotin [26]. Interpretation of antimicrobial susceptibility testing followed the Clinical and Laboratory Standards Institute (CLSI) guidelines, Performance Standards for Antimicrobial Susceptibility Testing (M100, 35th edition) [27].

For histological analysis, liver and muscle tissue samples from each fish were fixed in 10% neutral buffered formalin, dehydrated through graded ethanol solutions, embedded in paraffin, sectioned at 3–5 µm, stained with Carazzi hematoxylin and eosin, and examined using light microscopy.

For lipid visualization, additional liver samples were processed as frozen sections and stained with Sudan III and scarlet. Sections were briefly immersed in 50–70% ethanol, stained in a saturated alcoholic dye solution, rinsed, counterstained with hematoxylin, washed, and mounted in glycerogel.

Histological examination was performed using a Carl Zeiss Axioscope light microscope (Carl Zeiss Meditec, Jena, Germany). Digital images were captured using a Levenhuk camera (Levenhuk, Moscow, Russia) and ToupView software Legacy release version (ToupTek, Hangzhou, China). Histomorphometric analysis was conducted using OMERO v5.6, QuPath v0.5.1 [28], and Orbit Image Analysis v3.64 software [29]. Data processing and visualization were performed using the publicly available Cluster v2.1.4 package.

### 2.3. Studies to Determine CYPOR Concentrations in Liver Homogenates

Liver samples were homogenized in Tris–HCl buffer (pH 7.4) supplemented with 0.25 M sucrose, ethylenediaminetetraacetic acid (EDTA), and glutathione to chelate divalent metal ions and prevent lipid peroxidation. Homogenization was performed using a Stegler S10 homogenizer (Stegler, Moscow, Russia). CYPOR concentrations were determined by enzyme-linked immunosorbent assay (ELISA) using a StatFax 3200 analyzer (Awareness Technology, Palm City, FL, USA) and a Cytochrome P450 Reductase ELISA kit (Cloud-Clone Corp., Wuhan, China), according to the manufacturer’s instructions [30].

### 2.4. Statistical Analysis

The statistical analysis included calculation of the mean (M), median, standard deviation (SD), standard error (SE), and coefficient of variation. The standard deviation (SD) was calculated using the formula:

(1) $$SD=\sqrt{\frac{\Sigma_{i=1}^{n}{\left(x_{i}-\bar{x}\right)}^{2}}{n}}$$

where *x_i_*—i index; $\bar{x}$—arithmetic mean; *n*—number of samples.

The coefficient of variation (*CV*) was calculated using the formula:

(2) $$CV=\frac{SD}{\bar{x}}\cdot 100\%$$

where *SD*—standard deviation; $\bar{x}$—arithmetic mean.

The standard error (*SE*) was calculated using the formula:

(3) $$SE=\frac{SD}{\sqrt{n}}$$

where *SD*—standard deviation; *n*—number of samples.

Post hoc comparisons were performed using Student’s *t*-test for independent samples to evaluate groups in pairs, taking into account the Bonferroni correction. The calculation of these coefficients was performed in the software SATISTICA 10.0 (Dell Inc., Round Rock, TX, USA). Values for which *p* < 0.05 were considered significantly different. Three-dimensional graphical images were prepared using the Matplotlib 2.1.1 library and the Numpy 1.7.1 mathematical library [31].

## 3. Results

### 3.1. Ecological Status

The southern littoral zone of Lake Ladoga serves as a spawning and nursery area for the lake’s main fish species. The dominant negative influences in this region arise from numerous anthropogenic factors, including both organic and inorganic toxicants. Previous studies have shown that concentrations of high-molecular-weight compounds, particularly polychlorinated biphenyls (PCBs), significantly exceed maximum allowable concentrations (MACs) in these waters [32]. The concentrations of organochlorine compounds, including α- and γ-hexachlorocyclohexane (HCH), exceed permissible levels at almost all sampling points in Lake Ladoga and its tributaries. Lindane (γ-HCH), a particularly hazardous xenobiotic, has also been widely distributed throughout the lake.

Assessment of heavy metals and physical and hydrochemical parameters of water in three areas of Lake Ladoga was carried out during the summer seasons from 2022 to 2025, and the results are presented in Table 2.

The mineralization index ranged from 0.030 to 0.122 (station V1). Dissolved oxygen concentrations varied from 9.5 to 11.7 mg/L, with a lake-wide average of 10.4 mg/L. Oxygen saturation remained high, with an average of 102.2%. The oxidation–reduction potential (ORP) ranged from 43 to 156 mV, with a mean value of 96.5 mV, while pH values varied from 7.4 to 8.4, with an average of 7.6. These results are consistent with data reported by the Committee on Natural Resources of the Leningrad Region for the study period.

Fish studies were conducted in the littoral zones and adjacent waters of three bays: Shlisselburg, Volkhov, and Svir. For analysis, 10 sexually mature specimens of each species (bream, perch, roach, and pike-perch) were selected from each research station within the surveyed waters.

During external examination, most fish specimens from the southern part of the lake exhibited a body surface covered with a thin layer of transparent mucus without abnormal odor or turbidity. Gill appearance was carefully assessed: in some specimens, particularly from Volkhov Bay, anemia and hemorrhages of the gill tissue were observed. In cases of pronounced gill hemorrhaging, a musty or sour odor was detected. Mechanical damage and scale coverage were also evaluated. In most cases, scales were intact and firmly attached; however, some fish showed mechanical injuries and bruising. Corneal transparency and eyeball position relative to the orbit were assessed: freshly caught fish exhibited clear corneas and convex eyes. The abdomen was normal in most cases, while the anus appeared sunken and pale pink. The musculature was firm, with a shiny cross-sectional appearance characteristic of each species. Odor evaluation using a cooking test indicated freshness. No dangerous zoonotic helminths were detected in fish muscle tissue.

Some fish displayed poor body condition, fin tissue lesions, and other abnormalities. The spring survey revealed that lesions were consistent across all sampling areas and were associated with hemodynamic disturbances and focal degeneration in the liver.

Liver pathology included localized degenerative foci, alterations in organ color, and changes in consistency. Gallbladder distension and changes in bile color and consistency were observed. Cardiac examination revealed anemia and myocardial flaccidity. Kidneys were enlarged, congested, edematous, and occasionally showed renal tubular calculi. In the gastrointestinal tract, mucosal edema, hyperemia, and accumulation of mucus were noted. Vascular injection was observed in the gonads. The swim bladder exhibited congested vessels and occasional diffuse hemorrhages. Brain changes were characterized by vascular congestion and occasional pinpoint hemorrhages. Importantly, fish collected outside the littoral zone were generally in good condition.

The summer survey revealed a nearly identical intoxication pattern in both prevalence and severity. Fish with mild lesions predominated, and gill damage was generally less severe. Older fish and bottom-dwelling species were more strongly affected. Using bleak as an example, pelagic fish were shown to be less affected, with only mild intoxication.

Fish collected at monitoring stations exhibited predominantly mild intoxication. During the autumn survey of Shlisselburg Bay, fish condition was generally satisfactory, with mild pathological signs predominating. In contrast, spring surveys revealed occasional fish with poor body condition and clear signs of intoxication. Thus, springtime intoxication appears to be associated with overwintering stress and increased pollutant input via surface runoff and precipitation. Pathological examination data for fish from Volkhov Bay are presented in Table 3.

Fish in this area were collected both within and beyond the littoral zone. As shown in Table 3, multiple fish species were examined across different seasons.

**Figure 2:**
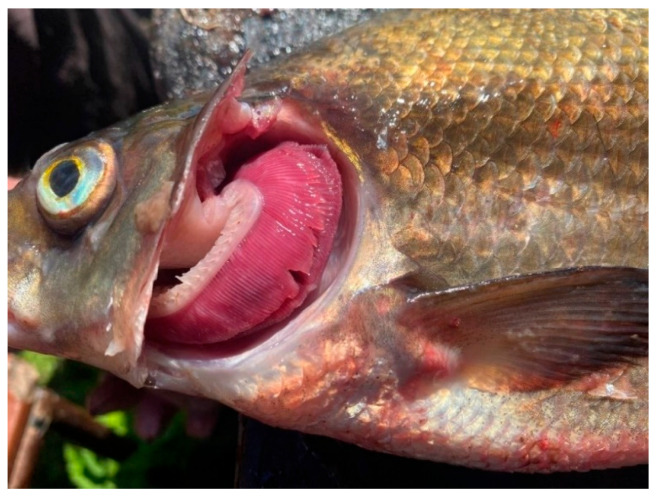
Anemia of fish gill tissue.

**Figure 3:**
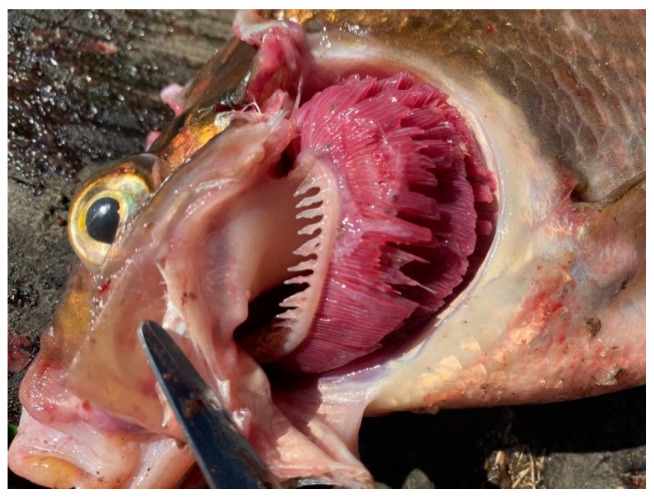
Pathology of fish gills: uneven coloration, soft tissue damage, excessive mucus buildup.

**Figure 4:**
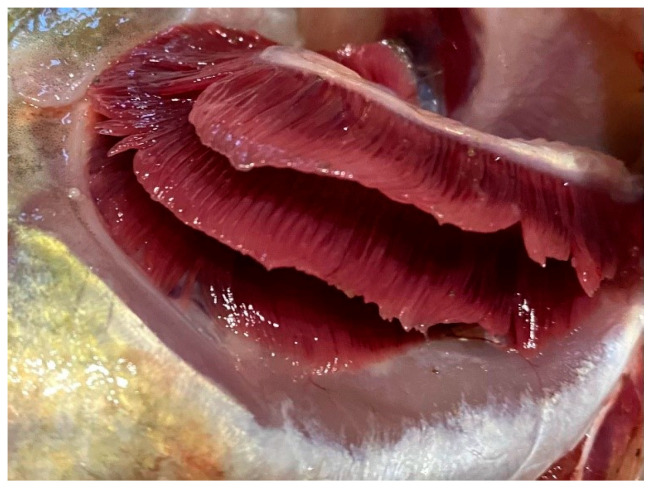
Pathology of fish gills: uneven coloration, presumably tissue necrosis regeneration.

**Figure 5:**
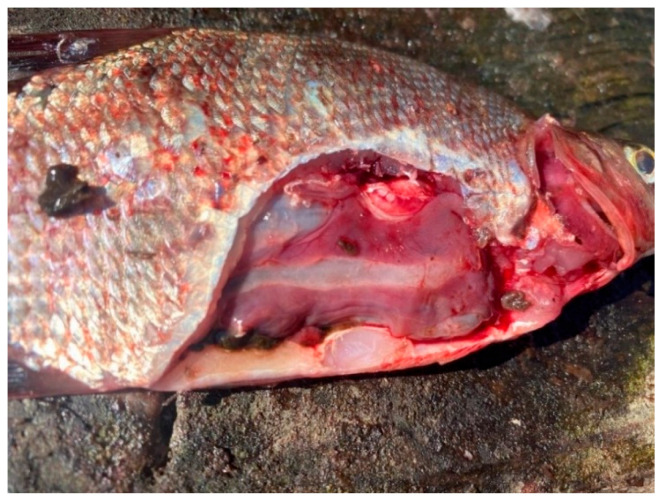
Bream liver in normal physiological condition.

**Figure 6:**
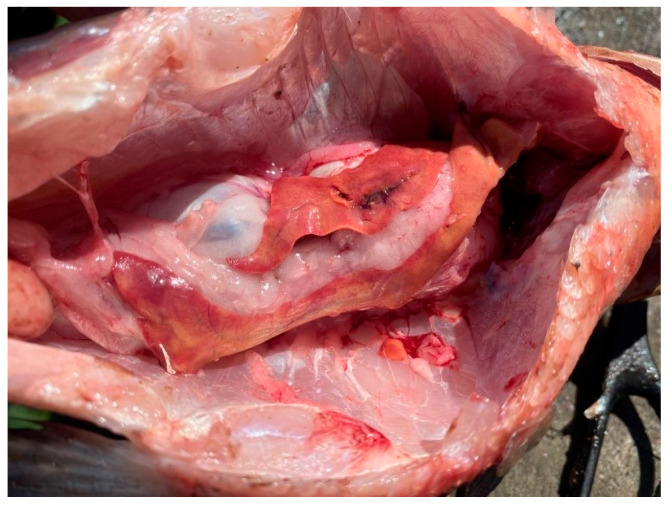
Fish liver pathology: liver degeneration.

**Figure 7:**
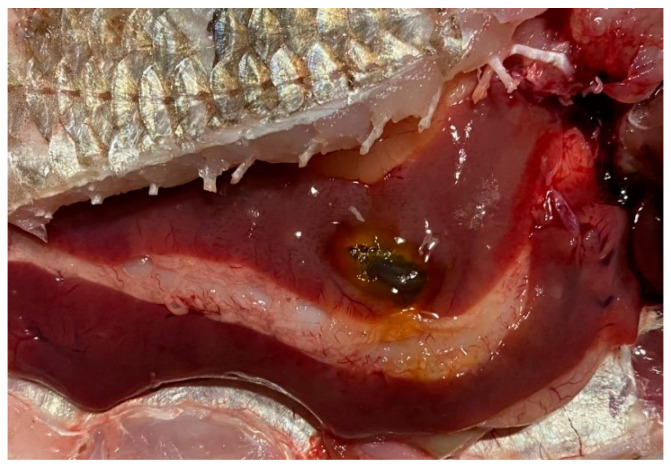
Fish liver pathology: hyperemia of the fish liver.

**Figure 8:**
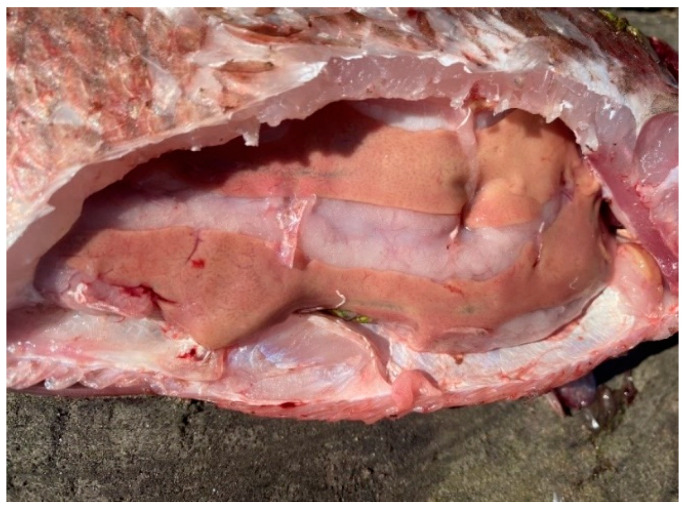
Pathology of fish liver: degeneration and deformation of the organ.

**Figure 9:**
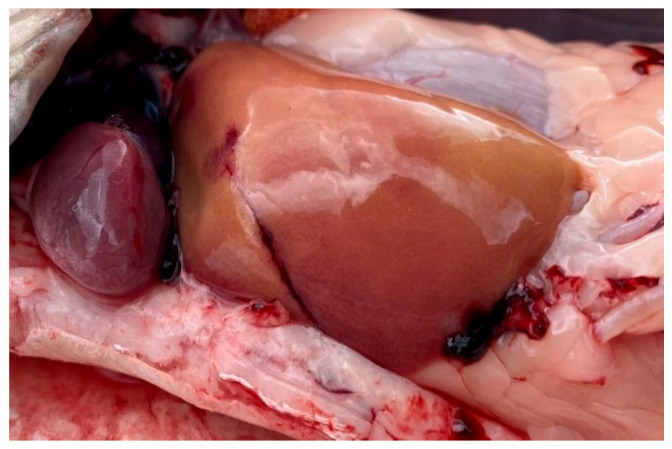
Pathology of fish liver: foci of degeneration.

Overall, the southern waters of Lake Ladoga, and especially the shallow littoral zone, judging by the state of fish, are the most polluted waters. The highest level of pollution was recorded in the Volkhov Bay of the lake. Pathological manifestations of chronic intoxication in fish from the southern part of the lake are shown in Figure 2, Figure 3, Figure 4, Figure 5, Figure 6, Figure 7, Figure 8 and Figure 9.

Pathomorphological examination of fish species from southern Lake Ladoga demonstrated that the observed pathological changes are characteristic of a chronic process.

The most pronounced lesions were observed in the gills. Histological analysis revealed disruption of the epithelial and lamellar architecture, vascular wall damage in necrotic regions, epithelial disintegration, and fusion of secondary lamellae into solid structures.

Liver pathology was characterized by pigmented lesions and lymphocytic infiltration among hepatocytes, which were frequently vacuolated and exhibited small, pigmented nuclei indicative of degenerative changes. Aggregates of pigment cells and lymphocytes were also present. Some fish exhibited hepatocyte discomplexation, connective tissue proliferation, and fibroblast accumulation around bile ducts and blood vessels.

In the kidneys, focal and diffuse hemorrhages, edema of glomeruli and convoluted tubules, and vacuolar–granular dystrophy of tubular epithelium were observed. These changes impair hemodynamics and filtration. Hemosiderin accumulation was detected in hematopoietic tissue, while connective tissue exhibited proteinaceous deposits. The most pronounced tubular degeneration occurred in fish from Volkhov Bay.

Brain tissue showed comparatively lower reactivity and a capacity for recovery. Germinal tissue appeared more resistant to toxic effects, suggesting high resilience of the reproductive system.

**Figure 10:**
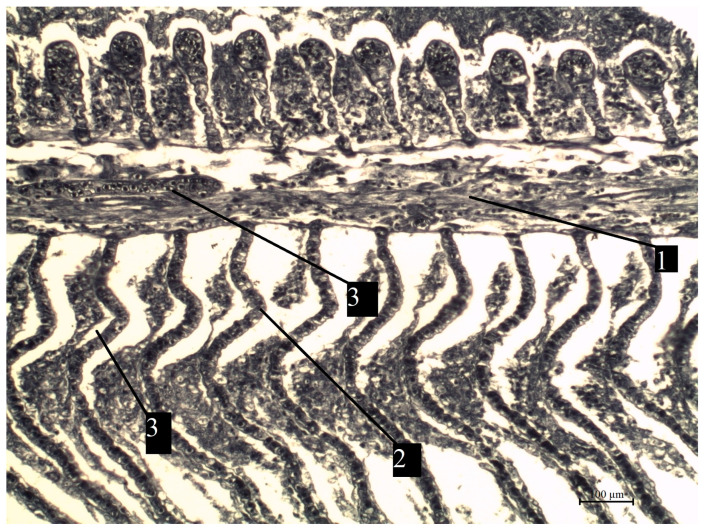
A fragment of the bream gills from the southern part of Ladoga Lake with preserved architecture. 1—petals (primary lamellae); 2—petals (secondary lamellae); 3—capillaries. Stained with iron hematoxylin according to Heidenhain, magnification: ×200.

**Figure 11:**
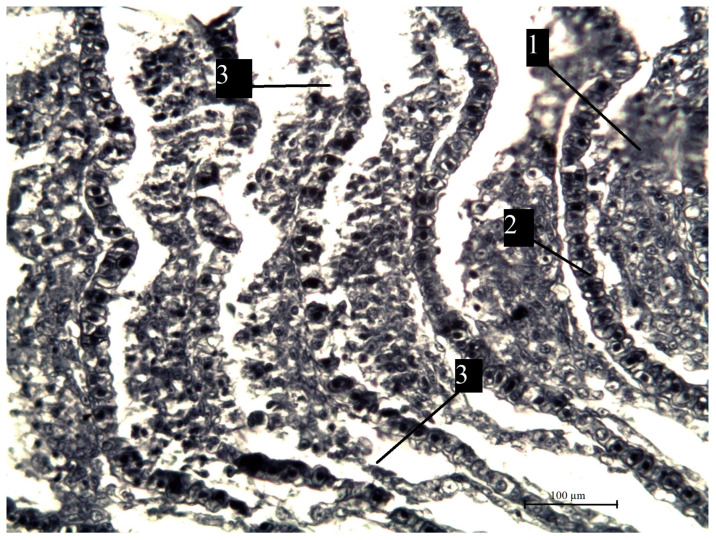
A fragment of bream gills from the southern part of Ladoga Lake with damaged blood vessels of the lamellae. 1—lobes (primary lamellae); 2—lobes (secondary lamellae); 3—damaged capillaries. Stained with iron hematoxylin according to Heidenhain, magnification ×200.

**Figure 12:**
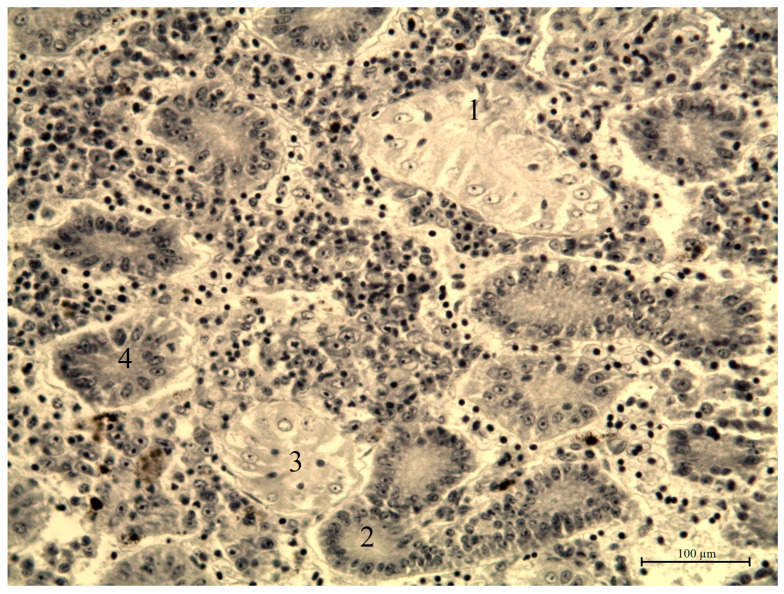
Granular dystrophy of the epithelial cells of the renal tubules of roach from the southern part of Ladoga Lake. 1—urinary collecting tube; 2—proximal convoluted tubule; 3—vascular glomerulus; 4—distal convoluted tubule. Stained with iron hematoxylin according to Heidenhain, magnification: ×200.

**Figure 13:**
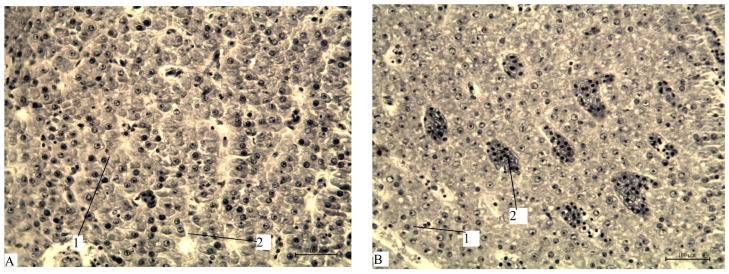
A fragment of the liver of bream (**A**) and perch (**B**) from the southern part of Ladoga Lake. (**A**) 1—hepatocyte nuclei; 2—central vein; (**B**) 1—blood vessel; 2—accumulation of macrophages containing melanin by Heidenhain’s hematoxylin, magnification: ×200.

**Figure 14:**
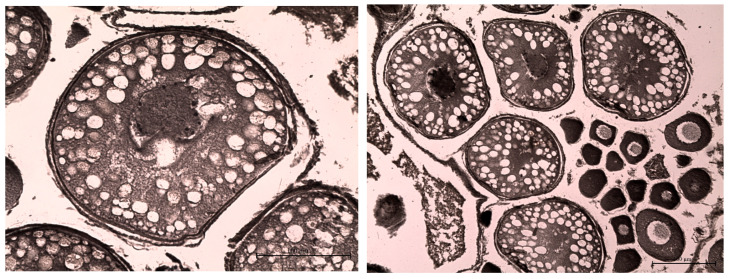
Vacuolization of older oocytes during vitellogenesis in perch from the southern part of Ladoga Lake. Iron hematoxylin staining according to Heidenhain, magnification: ×200.

Histopathological changes in internal organs and gills of fish from southern Lake Ladoga are shown in Figure 10, Figure 11, Figure 12, Figure 13 and Figure 14.

Studies have shown that the identified histomorphological abnormalities in the internal organs of fish and gill tissue in the southern waters of Lake Ladoga are widespread and are clear evidence of the impact of toxicological factors on the ichthyofauna of this water area, which is confirmed by the results of pathological and histological studies and the presence of visual manifestations of chronic intoxication.

### 3.2. Bacteriological Examination

During the bacteriological examination of pathological material isolated from fish, *Aeromonas* spp. and *Enterococcus* spp., which belong to opportunistic microflora, were isolated (Table 4).

*Aeromonas* spp. exhibited 100% resistance to chloramphenicol, gentamicin, polymyxin, and norfloxacin. *Enterococcus* spp. were 100% resistant to ampicillin, amoxicillin, chloramphenicol, gentamicin, and doxycycline.

*Aeromonas* spp. were detected in all examined fish, indicating their widespread distribution. While typical inhabitants of freshwater ecosystems, certain species can cause furunculosis, ulcers, and septicemia under stress conditions. *Enterococcus* spp. were detected in 55.6% of samples, primarily in intestinal contents, which is expected as part of normal gut microbiota. However, the high level of antibiotic resistance among wild strains is concerning. *Staphylococcus* spp. were also detected in 55.6% of samples, primarily on gills, and likely reflect external contamination.

Microbiological analysis of water samples revealed favorable characteristics, indicating suitability for fisheries. Results complied with sanitary and epidemiological standards outlined in Guidelines 4.2.1884-04 [33].

### 3.3. Hematological Examination Results

**Figure 15:**
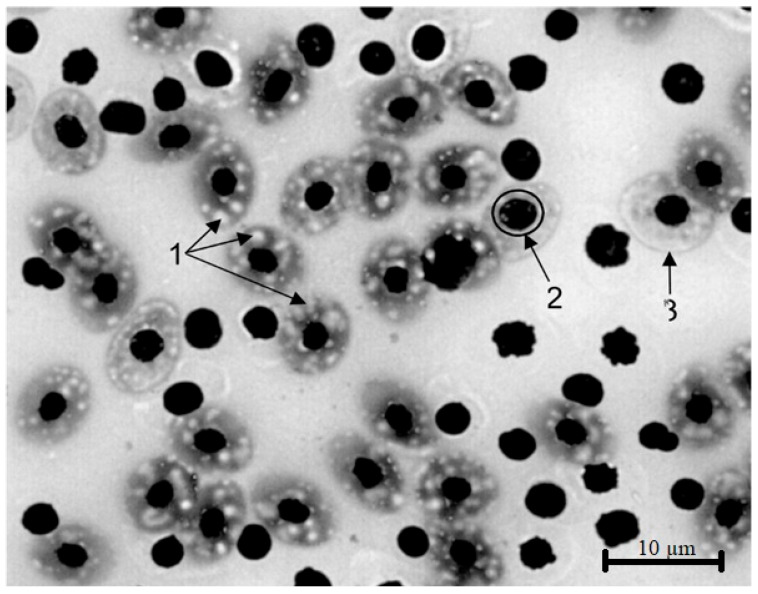
Microphotograph of a peripheral blood smear. 1—vacuolation of the erythrocyte cytoplasm, 2—vacuolation of the erythrocyte nucleus, 3—anisocytosis of erythrocytes. Stained according to Romanovsky. Magnification ×1000.

Microscopic analysis of peripheral blood smears from the studied fish (Figure 15) revealed significant ultrastructural abnormalities in erythrocytes. In the examined cell population, the proportion of cells with cytoplasmic vacuolization was 45.35%, indicating impaired degradation and recycling of cellular components. Vacuoles were diffusely distributed due to dysfunction of the lysosomal apparatus and accumulation of lipid inclusions. Nuclear vacuolization was also observed, which is characteristic of impaired nuclear–cytoplasmic transport. The coefficient of variation in erythrocyte diameter was 1.2, significantly exceeding the threshold value, indicating the presence of anisocytosis. Asynchrony of erythroid maturation may be a consequence of induced disruption of membrane structures and mitochondrial activity of erythroblasts against the background of reservoir contamination.

When assessing the cellular diversity of peripheral blood in the studied fish, it should be noted that the proportion of phagocytic cells with high migratory capacity—capable of actively absorbing not only bacteria but also products of cellular and tissue decay (profile metamyelocytes and monocytes)—in the leukogram exceeded reference values. This finding reflects intensification of the elimination of cellular destruction products induced by toxicants. The presence of lymphopenia combined with neutrophilia in blood smears represents a hematological marker of chronic intoxication in aquatic organisms and indicates prolonged exposure to aquatic contaminants.

### 3.4. Histological Examination

**Figure 16:**
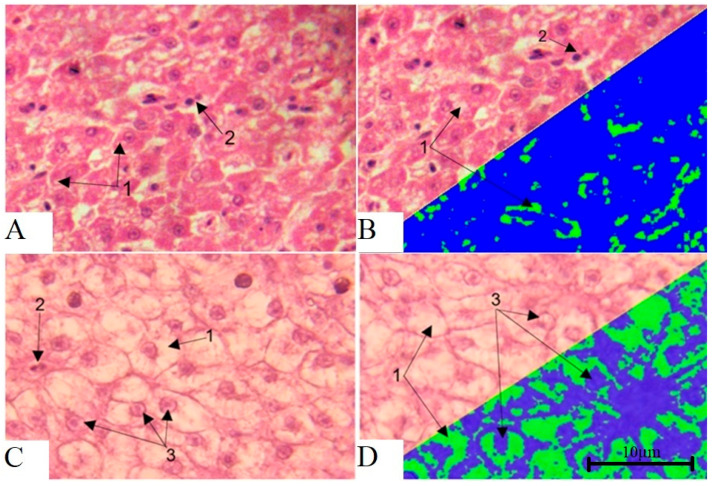
Micrographs of liver tissue. (**A**,**B**)—healthy fish, (**C**,**D**)—studied fish. 1—Vacuolization of the hepatocyte cytoplasm. 2—Binucleated cells. 3—Shift in nuclei to the periphery of hepatocytes. Stained with hematoxylin, Carazzi, and eosin. Magnification ×1000.

Histological examination of the liver of healthy fish (Figure 16A,B) revealed preservation of the classical organ architecture. Hepatocytes were characterized by a typical polygonal shape with distinct intercellular boundaries, and the cytoplasmic component was homogeneous. Hepatocyte nuclei exhibited a spherical configuration with uniform chromatin distribution. The presence of binucleated cells indicated regenerative activity of the organ. Moderate cytoplasmic granularity was observed. Sinusoidal spaces were clearly differentiated between hepatic trabeculae, and well-structured portal triads were visible in the peripheral regions of the liver lobules.

Histological examination of the liver in the studied fish (Figure 16C,D) revealed significant disorganization of the classical trabecular structure of the liver parenchyma, with disruption of the radial orientation of hepatocytes relative to the central veins in all 40 examined fish. Pronounced disintegration of tissue architecture was observed, accompanied by disruption of normal histotopographic relationships between cellular elements. Hepatocytes exhibited multiple pathological changes, including increased cell volume, decreased cytoplasmic optical density, and indistinct cytolemma, indicating disruption of osmotic homeostasis and intracellular fluid accumulation. In the centrilobular regions, pronounced degeneration of hepatocytes was observed, with total cytoplasmic vacuolization accounting for 56.4% of the cell area. Lipofuscin inclusions were detected in the cytoplasm of some hepatocytes, indicating chronic liver tissue damage caused by antimicrobial contamination. The presence of binucleated cells indicated ongoing regenerative activity. The nuclear apparatus of hepatocytes exhibited marked alterations, including peripheral displacement of nuclei due to compression by cytoplasmic inclusions and chromatin heterogeneity with areas of condensation.

**Figure 17:**
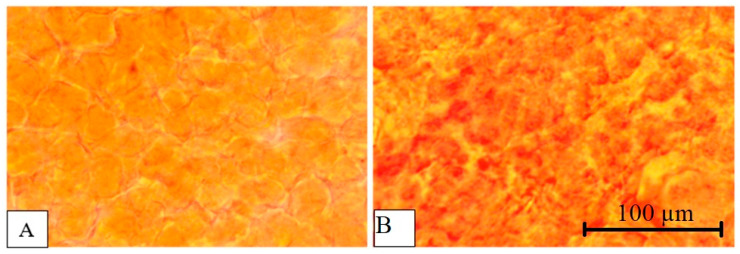
Micrographs of common carp (*Cyprinus carpio*) liver tissue. (**A**)—healthy specimens. (**B**)—specimens under study. Stained with Sudan III and scarlet red, magnification ×300.

To confirm the results obtained using Carazzi hematoxylin and aqueous–alcoholic eosin staining, histological examination with specific lipid staining using Sudan III and scarlet red was performed. Compared with healthy fish (Figure 17A), the livers of experimental fish (Figure 17B) demonstrated pronounced pathomorphological changes in the parenchyma consistent with fatty degeneration resulting from toxic injury, as evidenced by intense red–orange staining. Discomplexation of hepatic cords, disruption of the trabecular structure, and loss of clear intercellular boundaries were observed, reflecting hepatocyte hypertrophy due to excessive lipid accumulation. An increased karyocytoplasmic ratio, nuclear hyperchromia, and signs of karyopyknosis in individual cells were detected, indicating early necrobiotic changes. According to Kudersky [34], large lipid vacuoles form in the cytoplasm of hepatocytes under conditions of aquatic contamination, which is clearly demonstrated in the present specimens. These changes are accompanied by impairment of metabolic and detoxification liver functions, significantly reducing fish resistance to adverse environmental factors and potentially leading to mortality.

**Figure 18:**
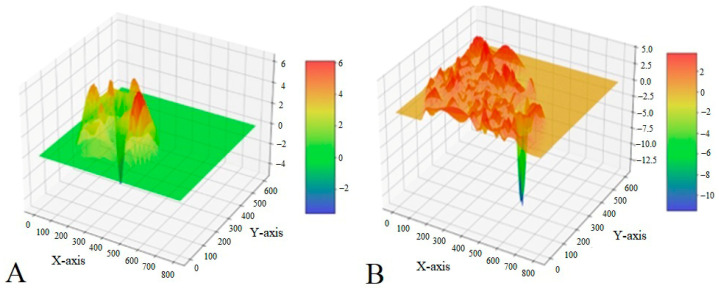
Three-dimensional mapping of the chromatic dispersion coefficient of lipid infiltration in the liver of healthy (**A**) and experimental (**B**) fish.

Three-dimensional graphical visualization of the chromatic dispersion coefficient of lipid infiltration clearly demonstrates structural differences between normal liver tissue of healthy fish and fatty-degenerated liver tissue of the studied fish (Figure 18). Figure 18A shows a predominantly uniform profile with dominant green coloration, consistent with preserved histoarchitecture of healthy liver tissue. In contrast, Figure 18B demonstrates a marked predominance of orange–red coloration and formation of numerous tall, irregular peaks. According to Zahran et al. [34], who used similar visualization techniques, the intensity of red coloration correlates with the degree of lipid accumulation.

Quantitative analysis of the presented surfaces revealed a significant increase in peak height and density in experimental fish, which, according to Pramanik and Biswas [35], reflects disorganization of hepatic beam structure and increased hepatocyte volume due to lipid vacuole accumulation. Enhanced surface relief also indicates inflammatory changes frequently associated with fatty degeneration, which is consistent with the findings reported by Rana [36].

**Figure 19:**
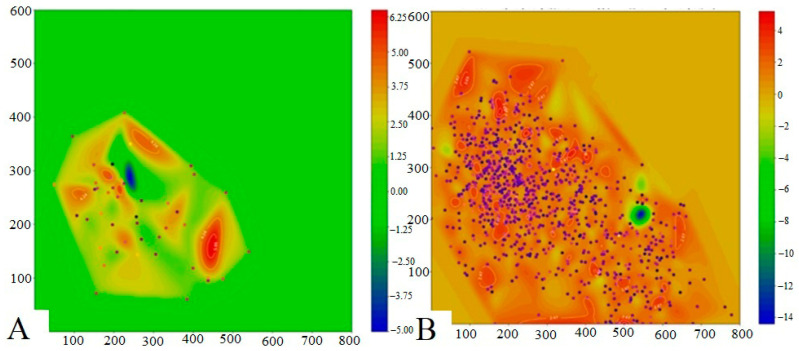
Heat map of the distribution of the chromatic dispersion coefficient of lipid infiltration of the liver of healthy (**A**) and experimental (**B**) fish. Purple points—maximum value of the chromatic dispersion coefficient.

The presented heat map of the chromatic dispersion coefficient distribution of lipid infiltration (Figure 19) complements and refines the data described above, providing a two-dimensional topographic visualization of the pathological process.

Healthy fish (Figure 19A) exhibited a predominantly uniform green background, with blue markers representing lipid droplets present in small quantities within several discrete foci. In contrast, the studied fish (Figure 19B) demonstrated a substantial increase in the number of blue markers diffusely distributed across the entire tissue section, indicating extensive fatty infiltration. Quantitative assessment showed that the liver lesion area in experimental fish exceeded 80% of the tissue, which, according to the classification of Pramanik and Biswas [35], corresponds to a severe degree of steatosis.

### 3.5. CYPOR Examination

To statistically evaluate differences in CYPOR levels between groups, one-way analysis of variance (ANOVA) was performed. The analysis revealed a highly significant main effect of environmental exposure on hepatic CYPOR concentration (F (3, 36) = 87.42, *p* < 0.001). In healthy fish, CYPOR concentrations ranged from 0.3 to 0.4 ng/mL, whereas in all examined fish from polluted areas, concentrations ranged from 5 to 6 ng/mL, confirming the influence of environmental factors on CYPOR expression. Post hoc comparisons using Tukey’s HSD test confirmed that CYPOR levels in fish from all polluted littoral zones (Shlisselburg, Volkhov, and Svir Bays) were significantly elevated (*p* < 0.001 for all comparisons) compared with the control group. No statistically significant differences in CYPOR concentrations were detected among the three polluted bays (*p* > 0.05), indicating a uniform stress response across the impacted littoral zone. The coefficient of variation (CV) was 8.7% in the control group and 22.4% in the combined exposed groups, reflecting increased variability of biomarker responses under natural, heterogeneous stress conditions.

This pronounced 15–20-fold increase in CYPOR activity in the experimental fish indicates strong induction of this enzyme by the studied environmental factors. Since CYPOR serves as an obligate electron donor for the cytochrome P450 system, such induction may substantially influence the metabolism of xenobiotics and endogenous compounds, potentially altering their biotransformation rates, toxicity, or pharmacological effects.

The obtained data suggest that exposure to these factors activates specific signaling pathways—likely mediated by AhR, PXR, or CAR receptors—that regulate transcription of the *POR* gene. A marked increase in CYPOR levels may reflect an adaptive response aimed at enhancing detoxification capacity; however, it also carries potential risks, including disruption of metabolic homeostasis, activation of procarcinogens, and altered drug efficacy.

Although the observed quantitative differences are substantial, further studies are required to fully elucidate their physiological consequences. Key directions for future research include:

1. Identification of specific factors (or their combination) responsible for induction;
2. Studying changes in the activity of CYPOR-dependent cytochrome P450 isoforms;
3. Assessment of the functional outcomes of such induction in vivo (e.g., clearance rate of specific substrates, formation of toxic metabolites, oxidative stress).

## 4. Discussion

The data obtained in this study reveal a profound contrast in cytochrome P450 reductase (CYPOR) levels between fish under controlled conditions and those from the anthropogenically influenced littoral zones of Lake Ladoga. The observed 15–20-fold increase in CYPOR in wild fish is a striking indicator of significant biochemical adaptation, consistent with findings in other polluted aquatic systems. For instance, studies on fish exposed to industrial effluents or urban runoff have reported similar upregulation of detoxification enzymes, including various CYP isoforms, as a generalized stress response [37]. However, the magnitude of CYPOR induction observed here is particularly notable and aligns with research highlighting its role as a central electron donor, potentially making it a more stable biomarker than individual, highly variable CYP isoforms.

Our integrated assessment—encompassing hydrochemical analysis, pathological examination, hematology, bacteriology, and CYPOR measurement—consistently points to a state of chronic physiological stress in fish from the southern littoral zone, particularly in Volkhov Bay. The observed pathological complex (gill lesions, hepatic fatty degeneration, renal tubular dystrophy, erythrocyte abnormalities) is characteristic of prolonged exposure to toxicants, mirroring findings in fish from other water bodies impacted by complex pollutant mixtures [13,35]. The concomitant induction of CYPOR aligns with the well-established role of this enzymatic system in responding to xenobiotic exposure [8,9]. This suggests an adaptive, albeit costly, upregulation of detoxification capacity, a phenomenon also documented in fish species like common carp (*Cyprinus carpio*) exposed to sub-lethal levels of agricultural or pharmaceutical pollutants [11,19].

A critical reading of our results necessitates explicitly stating their primary limitation: this study establishes strong correlations between poor ecological conditions, pathological states, and elevated CYPOR, but it does not provide direct causal evidence linking a specific pollutant to the observed effects. The lake’s contamination profile is complex, involving legacy pollutants like PCBs and HCH, potential antibiotic residues, and general anthropogenic eutrophication [32]. While “antimicrobial contamination” is discussed in the context of bacterial resistance and the scientific literature, our chemical analysis did not quantify antibiotic concentrations in water or tissues. Therefore, the narrative of “antimicrobial impact” remains a plausible hypothesis grounded in the observed antibiotic resistance patterns—a growing concern globally in aquatic environments [5,20]—and the known role of CYP in drug metabolism, rather than a proven causal pathway. The induction of CYPOR is a generalized response to a cocktail of stressors, including, but not exclusive to, pharmaceutical pollutants [3,10,17].

Our findings on antibiotic-resistant bacteria in seemingly healthy fish are concerning and support other studies indicating that aquatic environments act as reservoirs and mixing zones for resistance genes, even in the absence of overt disease [38]. This underscores an ecosystem-level impact of pollution beyond direct fish pathology.

A more synthetic analysis of our multi-method dataset allows us to propose a hierarchical interpretive model for the observed effects, which is supported by comparative ecotoxicological frameworks [35]:

Primary and Direct Indicators: Hydrochemical deviations and macroscopic pathological signs (gill hemorrhages, liver discoloration) serve as direct, albeit nonspecific, flags of habitat degradation, similar to baseline assessments used in many water quality monitoring programs.

Tissue-Level Confirmatory Indicators: Histopathological changes (hepatic steatosis, gill architecture disruption, renal degeneration) provide conclusive evidence of organ damage and are more sensitive than gross pathology. The severe fatty degeneration visualized via Sudan III staining and 3D mapping is a strong, direct marker of hepatotoxic injury, frequently reported in fish from contaminated sites [34].

Cellular and Subcellular Stress Responses: Hematological anomalies (anisocytosis, vacuolization) and significant CYPOR induction represent systemic and molecular-level adaptive responses. CYPOR elevation, in particular, emerges as a highly sensitive integrative biomarker. It reflects the overall “xenobiotic metabolic load” and the activation of conserved stress-response pathways (e.g., AhR, PXR), potentially offering a more robust and integrative signal than the expression levels of individual, highly inducible CYP isoforms, which can show greater temporal and interspecific variation [39].

This model posits that while pathological signs confirm damage, biomarkers like CYPOR can provide an early-warning signal of subclinical stress, potentially preceding overt tissue damage, a concept gaining traction in proactive environmental monitoring [36].

The value of this study extends beyond documenting pollution; it lies in outlining a pathway for more effective monitoring and focused research. The consistent spatial (littoral vs. pelagic) and temporal (seasonal) patterns in fish health and CYPOR levels identify the southern littoral, especially near point sources like the Syassky Pulp and Paper Mill, as a critical zone requiring prioritized remedial and monitoring actions, a management approach validated in other impacted watersheds.

To move from correlation to causation and refine risk assessment, future studies must:

- Conduct targeted chemical analyses to quantify specific pollutants (PCBs, PAHs, heavy metals, and a broad panel of pharmaceuticals) in water, sediments, and fish tissues from these high-risk sites.
- Correlate the concentrations of specific chemical groups with the magnitude of CYPOR induction and specific histopathological endpoints.
- Validate the biomarker utility of CYPOR by comparing its response with other established biomarkers (e.g., CYP1A activity via EROD assay, oxidative stress markers) in a controlled exposure experiment with key identified contaminants.
- Investigate the population-level consequences of this chronic stress by assessing reproductive health (gonadal histology, vitellogenin levels) and growth parameters in affected fish.

## 5. Conclusions

Based on the integrated analysis of ecological data, fish health, and molecular biomarkers, the following conclusions can be drawn:

- The littoral zones of southern Lake Ladoga, particularly Volkhov Bay, are subjected to significant anthropogenic pollution, leading to chronic toxic exposure in resident fish populations. This is evidenced by deteriorated water quality parameters, widespread histopathological damage in organs (liver, gills, kidneys), and hematological abnormalities.
- Fish from polluted sites exhibit a severe (15–20 fold) induction of hepatic cytochrome P450 reductase (CYPOR) compared to healthy controls. This dramatic upregulation is strongly correlated with the observed pathological state and environmental degradation.
- CYPOR demonstrates high potential as a sensitive and integrative biomarker for environmental stress in fish. Its response likely reflects the overall metabolic demand for detoxification imposed by complex pollutant mixtures in real-world ecosystems.
- The isolated fish microbiota, while typical for healthy individuals, exhibited concerning patterns of multi-antibiotic resistance, highlighting an additional risk associated with environmental contamination in Lake Ladoga.

The applied multi-disciplinary approach (hydrochemical, pathological, bacteriological, hematological, and enzymatic) provides an effective model for comprehensive ecosystem health assessment and identifies specific geographic areas requiring urgent remediation and continuous monitoring.

## Figures and Tables

**Table 1:** Depth at the research stations.

Water Area	Shlisselburg Bay				Volkhov Bay				Svir Bay			
Station	Sh1	Sh2	Sh3	Sh4 (C *)	V1	V2	V3	V4 (C *)	S1	S2	S3	S4 (C *)
Depth, m	8	3.5	5	15	6	6.3	6.5	10	7.1	8.3	6.4	20

* C—control stations.

**Table 2:** Physical and hydrochemical parameters of water in the southern part of Ladoga Lake.

Water Area	Station	h, m	Horizon	T, ℃	El, µS/cm	Mn, g/L	O_2_, mg/L	% Sat, (O_2_)	pH	Eh, mV
Volkhov Bay	V1	6	surface	19.1 ± 0.7	162.2 ± 0.5	0.103 ± 0.060	9.5 ± 0.5	103.2 ± 1.3	8.2 ± 0.4	133.0 ± 1.3
			bottom	19.8 ± 0.6	187.3 ± 0.6	0.122 ± 0.020	8.8 ± 0.3	95.5 ± 1.5	7.8 ± 0.3	80.0 ± 1.2
	V2	6.3	surface	19.4 ± 0.2	158.9 ± 0.9	0.103 ± 0.030	9.8 ± 0.7	106.2 ± 0.9	8.3 ± 0.3	43.0 ± 0.9
			bottom	18.6 ± 0.3	171.1 ± 0.7	0.115 ± 0.050	9.5 ± 0.5	101.3 ± 1.5	8.1 ± 0.4	62.0 ± 0.7
	V3	6.5	surface	16.7 ± 0.2	177.7 ± 0.5	0.067 ± 0.008	10.6 ± 0.3	110.5 ± 1.6	8.1 ± 0.5	71.0 ± 1.2
			bottom	15.8 ± 0.6	103.3 ± 0.2	0.066 ± 0.003	10.2 ± 0.1	106.7 ± 1.2	8.2 ± 0.5	83.0 ± 1.2
	V4	10	surface	18.6 ± 0.3	102.2 ± 0.9	0.114 ± 0.010	9.8 ± 0.6	103.5 ± 1.4	8.0 ± 0.6	66.0 ± 1.8
			bottom	17.4 ± 0.3	101.2 ± 0.7	0.095 ± 0.001	11.0 ± 0.4	96.5 ± 1.3	7.8 ± 0.2	84.0 ± 0.9
Svir Bay	S1	7.1	surface	12.8 ± 0.5	94.5 ± 0.8	0.061 ± 0.002	11.2 ± 0.3	105.7 ± 1.7	8.0 ± 0.1	99.0 ± 0.6
			bottom	12.0 ± 0.85	95.9 ± 0.6	0.060 ± 0.005	11.00 ± 0.1	106.6 ± 1.0	8.0 ± 0.3	112.0 ± 1.0
	S2	8.3	surface	12.8 ± 0.6	84.3 ± 0.8	0.056 ± 0.005	11.1 ± 0.4	105.5 ± 0.9	7.9 ± 0.5	90.0 ± 1.3
			bottom	12.6 ± 0.4	83.9 ± 0.7	0.055 ± 0.006	11.3 ± 0.2	105.5 ± 1.5	7.9 ± 0.2	125.0 ± 1.8
	S3	6.4	surface	15.8 ± 0.2	71.8 ± 0.6	0.032 ± 0.003	10.2 ± 0.3	102.3 ± 1.8	7.7 ± 0.3	109.0 ± 1.6
			bottom	15.5 ± 0.3	71.2 ± 0.5	0.030 ± 0.006	10.3 ± 0.5	100.8 ± 1.4	7.4 ± 0.2	130.0 ± 0.9
	S4	22	surface	9.7 ± 0.2	92.8 ± 0.7	0.060 ± 0.003	12.2 ± 0.6	105.8 ± 1.9	7.4 ± 0.5	131.5 ± 1.2
			bottom	9.5 ± 0.4	95.2 ± 0.2	0.060 ± 0.002	12.3 ± 0.5	102.6 ± 1.8	7.1 ± 0.4	110.0 ± 1.0
Shlisselburg Bay	Sh1	8	surface	16.0 ± 0.4	92.5 ± 0.5	0.061 ± 0.001	10.5 ± 0.1	105.8 ± 1.2	7.6 ± 0.4	84.2 ± 0.3
			bottom	14.5 ± 0.2	94.7 ± 0.5	0.061 ± 0.001	10.4 ± 0.2	98.7 ± 1.4	7.8 ± 0.4	156.2 ± 1.9
	Sh2	3.5	surface	14.5 ± 0.1	84.2 ± 0.5	0.053 ± 0.002	10.7 ± 0.2	105.5 ± 1.6	8.4 ± 0.1	70.5 ± 0.2
			bottom	13.0 ± 0.2	87.3 ± 0.4	0.053 ± 0.004	11.2 ± 0.3	105.5 ± 1.2	8.0 ± 0.5	92.5 ± 0.3
	Sh3	5	surface	11.2 ± 0.3	91.4 ± 1.2	0.055 ± 0.003	11.4 ± 0.3	103.0 ± 1.6	8.4 ± 0.1	87.0 ± 0.5
			bottom	10.2 ± 0.7	90.7 ± 0.5	0.063 ± 0.005	11.5 ± 0.4	98.9 ± 1.0	7.9 ± 0.1	98.3 ± 1.0
	Sh4	15	surface	10.3 ± 0.5	94.3 ± 1.0	0.065 ± 0.003	10.6 ± 0.2	101.6 ± 1.7	7.6 ± 0.7	96.0 ± 1.5
			bottom	10.0 ± 0.4	93.5 ± 0.7	0.066 ± 0.001	10.5 ± 0.9	97.7 ± 1.2	7.4 ± 0.6	95.0 ± 1.7

**Table 3:** Results of the seasonal study of the southern part of Ladoga Lake.

Seasons	Fishing Stations	Fish Species	Number of Fish Examined	Fish Condition Assessment		
				Number of Fish with Pathological Changes (%)	Pathology Severity (Points)	Number of Fish by Points
Spring	V1 5 km from the mouth of the Volkhov River	bream	10	50	2; 3; 4.0	2—2.0; 2—3.0; 1—4.0
		pike-perch	10	50	2; 3.0	2—2.0; 3—3.0
		roach	10	60	2; 3; 4.0	3—2.0; 2—3.0; 1—4.0
		perch	10	50	2; 3.0	2—2.0; 2—3.0; 1—4.0
	V2 Syassky Pulp and Paper Mill area	bream	10	60	2; 3.0	2—2.0; 4—3.0
		pike-perch	10	70	2; 3; 4.0	2—2.0; 4—3.0; 1—4.0
		roach	10	60	2; 3.0	2—2.0; 4—3.0
		perch	10	50	2; 3.0	2—2.0; 3—3.0
	V3 5 km to the left of the mouth of the Volkhov River	bream	10	50	2; 3.0	2—2.0; 3—3.0
		pike-perch	10	40	2; 3.0	2—2.0; 2—3.0
		roach	10	50	2; 3.0	2—2.0; 3—3.0
		perch	10	40	2; 3.0	2—2.0; 2—3.0
	V4 Outside the littoral zone	bream	10	40	2; 3.0	2—2.0; 2—3.0
		pike-perch	10	40	2; 3.0	2—2.0; 2—3.0
		perch	10	50	2; 3.0	1—2.0; 4—3.0
		smelt	10	30	2.0	3—2.0
Summer	V1 5 km from the mouth of the Volkhov River	bream	10	50	2; 3.0	2—2.0; 3—3.0
		pike-perch	10	60	2; 3.0	4—2.0; 2—3.0
		roach	10	50	2; 3.0	3—2.0; 2—3.0
		perch	10	50	2; 3.0	2—2.0; 3—3.0
	V2 Syassky Pulp and Paper Mill area	bream	10	60	2; 3; 4.0	2—2.0; 3—3.0; 1—4.0
		pike-perch	10	60	2; 3; 4.0	2—1.0; 4—3.0
		roach	10	50	2; 3.0	2—2.0; 3—3.0
		perch	10	60	2; 3.0	2—2.0; 4—3.0
	V3 5 km to the left of the mouth of the Volkhov River	bream	10	50	2; 3.0	2—2.0; 3—3.0
		pike-perch	10	40	2; 3.0	1—2.0; 3—3.0
		roach	10	50	2; 3.0	2—2.0; 3—3.0
		perch	10	40	2; 3.0	2—2.0; 2—3.0
	V4 Outside the littoral zone	bream	10	40	2; 3.0	2—2.0; 2—3.0
		pike perch	10	30	2; 3.0	2—2.0; 1—3.0
		roach	10	50	2; 3.0	2—2.0; 3—3.0
		smelt	10	30	2.0	3—2.0
Autumn	V1 5 km from the mouth of the Volkhov River	bream	10	40	2; 3.0	2—2.0; 2—3.0
		pike-perch	10	50	2; 3.0	3—2.0; 2—3.0
		roach	10	40	2; 3.0	2—2.0; 2—3.0
		perch	10	50	2; 3.0	2—2.0; 3—3.0
	V2 Syassky Pulp and Paper Mill area	bream	10	50	2; 3.0	3—2.0; 2—3.0
		pike-perch	10	60	2; 3.0	2—2.0; 4—3.0
		roach	10	50	2; 3.0	2—2.0; 3—3.0
		perch	10	50	2; 3.0	2—2.0; 3—3.0
	V3 5 km to the left of the mouth of the Volkhov River	bream	10	40	2—3.0	2—2.0; 2—3.0
		pike-perch	10	40	2—3.0	2—2.0; 2—3.0
		roach	10	30	2—3.0	2—2.0; 1—3.0
		perch	10	40	2—3.0	2—2.0; 2—3.0
	V4 Outside the littoral zone	bream	10	40	2—3.0	3—2.0; 1—3.0
		pike perch	10	30	2—3.0	2—2.0; 1—3.0
		roach	10	40	2—3.0	2—2.0; 2—3.0
		smelt	10	20	2.0	2—2.0

**Table 4:** Bacteriological examination of pathological material isolated from fish.

Pathological Material Tested	Bacteriological Examination Results
Sample No. 1	Isolated: *Aeromonas* spp. nonpathogenic to white mice (intestinal contents, gills), sensitive to chloramphenicol, gentamicin, polymyxin, and norfloxacin; resistant to ampicillin, amoxicillin, lincomycin, erythromycin, tetracycline, rifampicin, furazolidone, furadonin, enrofloxacin, tylosin, streptomycin, and doxycycline; *Enterococcus* spp. (intestinal contents), sensitive to ampicillin, amoxicillin, chloramphenicol, gentamicin, doxycycline; resistant to lincomycin, erythromycin, tetracycline, rifampicin, polymyxin, furazolidone, furadonin, norfloxacin, enrofloxacin, tylosin, streptomycin.
Sample No. 2	Isolated: *Aeromonas* spp. nonpathogenic to white mice (intestinal contents, gills), sensitive to chloramphenicol, gentamicin, polymyxin, and norfloxacin; resistant to ampicillin, amoxicillin, lincomycin, erythromycin, tetracycline, rifampicin, furazolidone, furadonin, enrofloxacin, tylosin, streptomycin, and doxycycline.
Sample No. 3	Isolated: *Aeromonas* spp. (gills) nonpathogenic to white mice, sensitive to chloramphenicol, gentamicin, polymyxin, norfloxacin; resistant to ampicillin, amoxicillin, lincomycin, erythromycin, tetracycline, rifampicin, furazolidone, furadonin, enrofloxacin, tylosin, streptomycin, doxycycline; *Enterococcus* spp. (intestinal contents), sensitive to ampicillin, amoxicillin, chloramphenicol, gentamicin, doxycycline; resistant to lincomycin, erythromycin, tetracycline, rifampicin, polymyxin, furazolidone, furadonin, norfloxacin, enrofloxacin, tylosin, streptomycin.
Sample No. 4	Isolated: *Aeromonas* spp. nonpathogenic for white mice (intestinal contents, gills), sensitive to chloramphenicol, gentamicin, polymyxin, norfloxacin; resistant to ampicillin, amoxicillin, lincomycin, erythromycin, tetracycline, rifampicin, furazolidone, furadonin, enrofloxacin, tylosin, streptomycin, doxycycline.

## Data Availability

The original contributions presented in this study are included in the article. Further inquiries can be directed to the corresponding author.

## Abbreviations

CYPOR	cytochrome P450 reductase
CYP	cytochrome P450
NADPH	reduced nicotinamide adenine dinucleotide phosphate
ROS	reactive oxygen species
PAHs	polycyclic aromatic hydrocarbons;
PCBs	polychlorinated biphenyls
ALT	Alanine Aminotransferase
AST	aspartate aminotransferase
PXR	pregnane X receptor
CAR	constitutive androstane receptor
AhR	aryl hydrocarbon receptor
Mn	mineralization
EC	electrical conductivity
ORP	oxidation-reduction potential
M	mean
SD	standard deviation
SE	standard error
CV	coefficient of variation;
MAC	Maximum Allowable Concentration
